# Association of tooth loss with liver cancer incidence and chronic liver disease mortality in a rural Chinese population

**DOI:** 10.1371/journal.pone.0203926

**Published:** 2018-09-17

**Authors:** Jake E. Thistle, Baiyu Yang, Jessica L. Petrick, Jin-Hu Fan, You-Lin Qiao, Christian C. Abnet, Philip R. Taylor, Katherine A. McGlynn

**Affiliations:** 1 Division of Cancer Epidemiology and Genetics, National Cancer Institute, NIH, Rockville, Maryland, United States of America; 2 Department of Cancer Epidemiology, Cancer Institute/Hospital, Chinese Academy of Medical Sciences & Peking Union Medical College, Beijing, People’s Republic of China; Tufts University, UNITED STATES

## Abstract

**Background:**

Tooth loss has been reported to be associated with the risk of liver cancer in several prior studies in economically advantaged countries. Whether this relationship is also evident in economically disadvantaged populations is not known.

**Methods:**

We analyzed data from the Nutrition Intervention Trials, two randomized placebo-controlled trials of vitamin/mineral supplementation in Linxian, China. Participants who reported having lost permanent teeth were examined to determine the number of teeth remaining. In the 30-year follow-up period, 329 liver cancers were diagnosed and 368 chronic liver disease deaths occurred. Tooth loss was categorized based on loess smoothed age-specific predicted quartiles. Cox proportional hazards regression was used to estimate hazard ratios (HRs) and 95% confidence intervals (CIs) for the two outcomes.

**Results:**

Overall, persons in the highest quartile of age-specific tooth loss had an increased risk of liver cancer (HR = 1.27, 95%CI: 0.96, 1.67) which was not statistically significant. Results varied by sex and body mass index (BMI), however. Women in the highest quartile of age-specific tooth loss had a significantly increased risk (HR = 1.64, 95%CI: 1.04, 2.59), while men did not (HR = 1.08, 95%CI = 0.75, 1.57), and persons with a BMI > 23.0 kg/m^2^ (HR = 1.71, 95%CI: 1.00, 2.92) had a significantly increased risk, while persons with a BMI <23.0 kg/m^2^ did not (HR = 1.14, 95%CI: 0.82, 1.5). No relationships with chronic liver disease mortality were observed.

**Conclusions:**

In a rural, economically disadvantaged population, persons with the highest levels of age-specific tooth loss had an increased risk of liver cancer. The results, which were stronger among women and persons with greater BMI, suggest that periodontal disease may increase risk of liver cancer.

## Introduction

Liver cancer is the sixth most commonly occurring cancer in the world and the second leading cause of cancer mortality [1]. All major liver cancer risk factors cause chronic inflammation, which may progress to chronic liver disease and eventually liver cancer. Liver disease, particularly its most severe form, cirrhosis, is itself a major source of mortality [2]. The great majority of liver cancers occur in either Africa or Asia, with China alone accounting for half of all cases worldwide [3]. In China, major risk factors include consumption of aflatoxin-contaminated food and chronic hepatitis B and C virus (HBV/HCV) infection [4].

A factor that may be related to risk of liver cancer is periodontal disease, a common condition common among adults [5], with rising prevalence in China [6], that results from infection of the tissue surrounding and supporting the teeth. Severe disease, called periodontitis, includes destruction of connective tissue, bone loss, and tooth loss. Periodontitis may have systemic health effects as evidenced by studies of cardiovascular disease [7], and stroke [8], and has been associated with cancers of the mouth [9], esophagus [10, 11], and gastrointestinal tract [11, 12].

Using tooth loss as a proxy variable for periodontal disease, three prior studies have prospectively evaluated the association between tooth loss and risk of liver cancer [13–15]. A cohort study of male Finnish smokers [15] and a hospital case-control study in Japan [14] both reported increased risks of liver cancer with higher levels of tooth loss. In contrast, a survey of octogenarians in Fukuoka, Japan found no association between tooth loss and liver cancer [13]. As the results of these studies varied, and two of the studies relied on self-report of tooth loss rather than on dental examination, we sought to evaluate the relationship of tooth loss and liver cancer in a rural, economically disadvantaged population in which tooth loss was determined by dental exam.

## Materials and methods

### Study population

The Nutrition Intervention Trials were two randomized placebo-controlled trials of vitamin/mineral supplementation in Linxian, China, a rural county with high rates of esophageal and gastric cancers. Both the General Population Trial [16] and the Dysplasia Trial [17], have been previously described [18]. Briefly, the General Population Trial [16] consisted of 29,584 persons aged 40–69 at baseline who were randomized to intervention groups receiving daily multivitamin/mineral supplementation starting on March 1986. The Dysplasia Trial [17] consisted of 3,318 persons aged 40–69 with cytological evidence of esophageal dysplasia who were randomized to intervention with multivitamin/mineral supplements starting in May 1985. Data from the two trials were pooled and participants were excluded from the analysis if they did not provide questionnaire information about permanent teeth loss (n = 126), if they did not receive an examination after reporting any permanent teeth loss (n = 80), or if their recorded censoring date occurred before the start of intervention (n = 7). The final analytic population consisted of 32,689 participants.

The study protocols were approved by the Institutional Review Board of the National Cancer Institute. The study was registered as ClinicalTrials.gov number NCT00342654.

### Data collection

#### Conventional tooth loss

At the time of study recruitment, participants completed a baseline questionnaire and received a dental examination. Participants who reported, via questionnaire, having lost permanent teeth were examined to determine the number of teeth remaining. Number of teeth lost was categorized into the following groups: 0, 1–3, 4–6, 7–10, 11–20, and 21–32 teeth lost.

#### Age-specific tooth loss

As extent of tooth loss is positively related to age, loess smoothed age-specific predicted values for number of teeth lost were used for risk calculations [19]. The median tooth loss for each age was determined and loess smoothing was used to predict polynomial age-specific medians and quartiles over the range of ages. Persons were categorized into a four-category variable of quartiles.

#### Liver cancer incidence and liver disease mortality

In the trial (1985–1991) and post-trial (1991 onward) follow-up periods, incident cancer cases and causes of death were identified using several methods that captured near complete ascertainment. In the trial period, village health workers visited all participants, and trial staff reviewed records at all local and regional hospitals and the local cancer registry on a monthly basis. New cancer diagnoses and causes of mortality were confirmed by a panel of American and Chinese experts. In the post-trial period, village health workers continued monthly visits, with all new cancer diagnoses and causes of mortality confirmed by a panel of Chinese experts. These expert reviews consisted of review of diagnostic material, including pathology and cytology slides, ultrasonography reports, computerized tomography scans, clinical histories, biochemical results, and endoscopy and surgery reports.

Diagnoses of incident liver cancer were based on biochemical assays, clinical examination, ultrasound, and computerized tomography scans. Chronic liver disease mortality, including deaths due to cirrhosis, were identified by symptoms (jaundice, ascites, bruising and bleeding, palmar erythema, gynecomastia and hypogonadism), biochemical assays and computerized tomography scans. As of March 31^st^, 2016, 329 incident liver cancers were diagnosed and 368 chronic liver disease deaths had occurred.

#### Covariates

Potential covariates, specified *a priori*, were age at study randomization, sex, body mass index (BMI), education, cigarette smoking, alcohol use and study trial (General Population vs. Dysplasia). Age was self-reported at the time of start of the intervention and analyzed as a continuous variable. BMI was calculated from the measured height and the measured weight and categorized based on World Health Organization recommendations for Asian populations [20] into the following groups: < 18.50 kg/m^2^, 18.50–22.99 kg/m^2^, 23.00–27.49 kg/m^2^, and ≥ 27.50 kg/m^2^. Education was categorized into the following groups: no formal school, 1–5 years of education, primary school, middle school or higher, and other. Smoking status was categorized into never, former, and current smoking. Alcohol use was categorized as none vs. any drinking in the previous 12 months. Diabetes was examined as a potential covariate; however, positive diabetes status was self-reported by only 0.1% of the study population. Positive hepatitis status, self-reported by 3.6% of the study population, was also examined as a potential covariate, however, as the term ‘hepatitis’ lacks specificity, it was not included in the models. As the exposures were unrelated to trial intervention assignments, the models only adjusted for trial participation, rather than trial intervention.

#### Population subsample with HBV/HCV status

Blood samples were collected from participants at baseline in both trials. Serum markers to determine HBV and HCV infection status were analyzed in two previous nested case-controls studies in the Linxian trials population [21, 22]. Antibody to hepatitis C virus (anti-HCV) was analyzed using the ORTHO HCV version 3.0 enzyme-linked immunosorbent assay (ELISA) Test System from Ortho-Clinical Diagnostics, Raritan, NJ; hepatitis B surface antigen (HBsAg) was analyzed by enzyme immunoassay using the Bio-Rad Genetic Systems HBsAg EIA 3.0 kit of Bio-Rad Laboratories, Hercules, CA; and antibody to hepatitis B core antigen (anti-HBc) was analyzed by ELISA using the HBc (recombinant) ORTHO ELISA Test System of Ortho-Clinical Diagnostics.

### Statistical analysis

Follow up time began at the start of intervention (March 1, 1986 for General Population Trial and May 1, 1985 for Dysplasia Trial) and ended with the first diagnosis of liver cancer, death (from any cause), or March 31, 2016, resulting in 650,235 person-years of follow up. The frequency and proportion of covariates were calculated overall and within categories of age-specific tooth loss. Cox proportional hazards regression was used to estimate the hazard ratio (HR) and 95% confidence intervals (CIs) for the two outcomes, liver cancer incidence and chronic liver disease mortality. For conventional tooth loss, three methods to were compared to control for age: adjustment for age at randomization and age^2^ as continuous variables; stratification using 1-year age groups; and stratification using 5-year age groups. No meaningful difference was seen among these methods, thus age at randomization and age^2^ were used in all models for conventional tooth loss. All p-values were two-sided with a significance level of α = 0.05.

Measures of association were estimated with adjustment for age at randomization and age^2^ using loess smoothed age-specific predicted cut-offs, sex, BMI, education, cigarette smoking, alcohol use, and study trial. Tests for trend were constructed using either the number of teeth lost or the quartile of age-specific tooth loss as continuous variables. The proportional hazards assumption was tested using an interaction between tooth loss and log (follow-up time), as a continuous variable, in models that included adjustment for confounding. No violations of the assumptions were observed. The following sensitivity analyses were performed to assess the robustness of the findings: (1) Restricted to participants ≥ 50 years old at randomization, as periodontal disease is the main cause of tooth loss in older populations; (2) Restricted to persons who never smoked to address concerns of confounding by cigarette smoking [23]; (3) Excluded the first two years of follow-up to reduce the influence of preclinical, undiagnosed liver disease causing tooth loss; (4) Restricted to participants with known HBV/HCV status (n = 1,644); (5) Excluded the participants in the Dysplasia Trial. In addition, stratified analysis by sex and BMI (< 23.0 kg/m^2^ vs. ≥ 23.0 kg/m^2^) were conducted, based on recent animal studies of sex differences in inflammatory markers [24, 25] and studies of the contribution of metabolic syndrome to the periodontal-hepatic relationship [26, 27]. All analyses were performed using SAS 9.3 (SAS Institute Inc., Cary, NC).

## Results

Demographic characteristics of the study population by age-specific tooth loss quartile are shown in **Table 1**. The majority of the study population had a BMI of 18.5–22.99 kg/m^2^ (65.7%), had less than a primary school education (71.4%), were non-smokers (70.1%) and did not consume alcohol (77.0%).

**Table 1:** Characteristics of study participants by age-specific tooth loss quartile in the Nutrition Intervention Trials Cohort, Linxian, PRC, 1986–2016.

				Age-specific tooth loss quartile^a^							
				I		II		III		IV	
		Total		No. (%)		No. (%)		No. (%)		No. (%)	
Incident liver cancer											
	Yes	370	(1.1)	114	(1.1)	92	(1.3)	64	(0.9)	100	(1.2)
	No	32319	(98.9)	10210	(98.9)	6959	(98.7)	6689	(99.1)	8461	(98.8)
Chronic liver disease mortality											
	Yes	408	(1.2)	120	(1.2)	95	(1.3)	87	(1.3)	106	(1.2)
	No	32281	(98.8)	10204	(98.8)	6956	(98.7)	6666	(98.7)	8455	(98.8)
Age at randomization											
	<44	7911	(24.2)	3834	(37.1)	588	(8.3)	1740	(25.8)	1749	(20.4)
	45–49	5445	(16.7)	1690	(16.4)	1283	(18.2)	958	(14.2)	1514	(17.7)
	50–54	5968	(18.3)	1537	(14.9)	1634	(23.2)	1140	(16.9)	1657	(19.4)
	55–59	5687	(17.4)	1401	(13.6)	1556	(22.1)	1139	(16.9)	1591	(18.6)
	60–54	4565	(14.0)	1099	(10.6)	1178	(16.7)	1016	(15.0)	1272	(14.9)
	≥65	3113	(9.5)	763	(7.4)	812	(11.5)	760	(11.3)	778	(9.1)
Sex											
	Male	14541	(44.5)	5522	(53.5)	3397	(48.2)	2726	(40.4)	2896	(33.8)
	Female	18148	(55.5)	4802	(46.5)	3654	(51.8)	4027	(59.6)	5665	(66.2)
BMI (kg/m^2^)											
	< 18.50	2339	(7.2)	493	(4.8)	422	(6.0)	545	(8.1)	879	(10.3)
	18.50–22.99	21474	(65.7)	6667	(64.6)	4689	(66.5)	4424	(65.5)	5694	(66.5)
	23.00–27.49	8130	(24.9)	2902	(28.1)	1750	(24.8)	1636	(24.2)	1842	(21.5)
	≥ 27.5	743	(2.3)	262	(2.5)	187	(2.7)	148	(2.2)	146	(1.7)
	Missing	0		0		3		0		0	
Education											
	No formal school	13195	(40.4)	2980	(28.9)	3144	(44.6)	2921	(43.3)	4150	(48.5)
	1–5 years of education	10137	(31.0)	3575	(34.6)	2180	(30.9)	1997	(29.6)	2385	(27.9)
	Completed primary school	3415	(10.4)	1473	(14.3)	549	(7.8)	690	(10.2)	703	(8.2)
	Middle school or higher	2857	(8.7)	1512	(14.6)	357	(5.1)	525	(7.8)	434	(5.1)
	Other	3080	(9.4)	784	(7.6)	790	(11.2)	619	(9.2)	887	(10.4)
	Missing	0		0		2		1		2	
Cigarette smoking											
	Never	22915	(70.1)	6886	(66.7)	4729	(67.1)	4848	(71.8)	6452	(75.4)
	Former	647	(2.0)	238	(2.3)	164	(2.3)	116	(1.7)	129	(1.5)
	Current	9126	(27.9)	3200	(31.0)	2158	(30.6)	1789	(26.5)	1979	(23.1)
	Missing	0		0		0		0		1	
Alcohol use											
	Yes	7509	(23.0)	2826	(27.4)	1604	(22.8)	1498	(22.2)	1581	(18.5)
	No	25179	(77.0)	7498	(72.6)	5446	(77.2)	5255	(77.8)	6980	(81.5)
	Missing	0		0		1		0		0	
Study trial											
	General Population	29399	(89.9)	9638	(93.4)	6467	(91.7)	6028	(89.3)	7266	(84.9)
	Dysplasia	3290	(10.1)	686	(6.6)	584	(8.3)	725	(10.7)	1295	(15.1)
HBV/HCV status^b^											
	anti-HBc(+)	1048	(63.7)	320	(65.6)	233	(59.6)	215	(64.4)	280	(65.0)
	HBsAg(+)	214	(13.0)	75	(15.4)	39	(10.0)	45	(13.5)	55	(12.8)
	anti-HCV(+)	160	(9.7)	62	(12.7)	32	(8.2)	20	(6.0)	46	(10.7)

BMI = body mass index; anti-HBc = antibody to hepatitis B virus core antigen; HBsAg = hepatitis B virus surface antigen; anti-HCV = antibody to HCV

^a^Age-specific quartiles of tooth loss were estimated using loess regression

^b^Determined among 1644 patients free of liver cancer and chronic liver disease mortality selected in two case-controls studies nested in this population

**Table 2** shows the adjusted association of tooth loss with incident liver cancer and chronic liver disease mortality. In regard to liver cancer, there was no association with ever/never loss of permanent teeth (HR = 1.10, 95% CI: 0.84, 1.42) or with number of teeth lost (p_trend_ = 0.22). Persons in the highest quartile of age-specific tooth loss, however, had an increased risk of liver cancer (HR = 1.27, 95%CI: 0.96, 1.67) although the result did not attain statistical significance. With regard to chronic liver disease mortality, there were no significant associations with ever/never loss of teeth (HR = 1.01, 95% CI: 0.78, 1.29), number of teeth lost (p_trend_ = 0.33), or with age-specific tooth loss in the highest quartile (HR = 1.13, 95%CI: 0.86, 1.48).

**Table 2:** Association of tooth loss to incident liver cancer and chronic liver disease (CLD) mortality in the Nutrition Intervention Trials Cohort, Linxian, China, 1986–2016.

			Incident liver cancer			Chronic liver disease mortality		
		Person-years	N	HR^a^	95% CI	N	HR^a^	95% CI
Ever lost a tooth		** **	** **					
	No	187,621	87	1.00	referent	62	1.00	referent
	Yes	464,413	283	1.10	(0.84, 1.42)	228	1.01	(0.78, 1.29)
Number of teeth lost		** **	** **	** **	** **	** **	** **	** **
	0	187,621	87	1.00	referent	62	1.00	referent
	1–3	81,929	43	1.05	(0.73, 1.51)	27	0.74	(0.49, 1.10)
	4–6	107,152	58	1.06	(0.76, 1.49)	36	0.92	(0.65, 1.28)
	7–10	88,568	50	1.05	(0.73, 1.51)	51	1.24	(0.89, 1.71)
	11–20	93,649	61	1.15	(0.80, 1.64)	51	1.15	(0.83, 1.60)
	21–32	93,116	71	1.25	(0.87, 1.79)	63	1.10	(0.78, 1.55)
* *	*p-value for trend*			*0*.*22*			*0*.*33*	
Age-specific tooth loss^b^								
	I	222,428	114	1.00	referent	81	1.00	referent
	II	130,475	92	1.20	(0.90, 1.58)	65	1.08	(0.82, 1.42)
	III	133,181	64	0.97	(0.71, 1.32)	67	1.14	(0.86, 1.51)
	IV	165,951	100	1.27	(0.96, 1.67)	77	1.13	(0.86, 1.48)
* *	*p-value for trend*			*0*.*22*			*0*.*33*	

HR = hazard ratio; CI = confidence interval

^a^Estimated using cox-proportional hazards regression adjusted for age (age (at randomization) and age2 or loess smoothed age-specific predicted quartiles) gender, body mass index, body mass index, education, cigarette smoking, alcohol use, and trial membership

^b^Age-specific quartiles of tooth loss were estimated using loess regression; p-value for trend using either the number of teeth loss or the quartile of age-specific tooth loss as a continuous covariate

**Table 3** displays the results of analyses stratified on sex. Although there was no indication of interaction by sex (p_interaction_ = 0.67), the stratified analyses found women with the greatest number of teeth lost had a significantly increased risk of liver cancer (HR = 1.78, 95%CI: 1.00, 3.18) while the men did not (HR = 0.99, 95%CI: 0.61, 1.59). Similarly, women in the highest quartile of tooth loss had a significantly increased risk of liver cancer (HR = 1.64, 95%CI: 1.04, 2.59) while the men did not (HR = 1.08, 95%CI: .075, 1.57). There were no associations with chronic liver disease mortality among either men or women.

**Table 3:** Association of tooth loss to incident liver cancer and chronic liver disease (CLD) mortality by gender in the Nutrition Intervention Trials Cohort, Linxian, China, 1986–2016.

		Male							Female						
			Liver cancer incidence			CLD mortality				Liver cancer incidence			CLD mortality		
		Person-yrs	N	HR^a^	95% CI	N	HR^a^	95% CI	Person-yrs	N	HR^a^	95% CI	N	HR^a^	95% CI
Ever lost a tooth		** **	** **							** **	** **				
	No	91,668	62	1.00	referent	55	1.00	referent	95,953	25	1.00	referent	37	1.00	referent
	Yes	175,768	159	0.98	(0.71, 1.34)	145	0.96	(0.69, 1.35)	288,645	124	1.35	(0.86, 2.13)	171	1.05	(0.72, 1.53)
Number of teeth lost		** **	** **	** **	** **	** **	** **	** **	** **	** **	** **	** **	** **	** **	** **
	0	91,668	62	1.00	referent	55	1.00	referent	95,953	25	1.00	referent	37	1.00	referent
	1–3	38,776	29	0.97	(0.62, 1.51)	20	0.74	(0.44, 1.25)	43,153	14	1.20	(0.62, 2.31)	13	0.72	(0.38, 1.36)
	4–6	45,017	34	0.92	(0.60, 1.42)	34	1.00	(0.65, 1.55)	62,135	24	1.37	(0.78, 2.42)	23	0.80	(0.47, 1.36)
	7–10	32,133	31	1.02	(0.65, 1.60)	35	1.27	(0.82, 1.99)	56,435	19	1.14	(0.62, 2.10)	35	1.19	(0.74, 1.92)
	11–20	30,108	32	1.02	(0.65, 1.63)	26	0.90	(0.54, 1.48)	63,541	29	1.43	(0.80, 2.54)	50	1.34	(0.84, 2.11)
	21–32	29,734	33	0.99	(0.61, 1.59)	30	0.94	(0.57, 1.55)	63,382	38	1.78	(1.00, 3.18)	50	1.24	(0.76, 2.00)
* *	*p-value for trend*^*b*^	* *	* *	*0*.*80*	* *	* *	*0*.*89*	* *	* *	* *	*0*.*09*	* *	* *	*0*.*17*	* *
Age-specific tooth loss^c^															
	I	111,824	85	1.00	referent	78	1.00	referent	110,604	29	1.00	referent	42	1.00	referent
	II	57,851	55	1.04	(0.74, 1.47)	48	0.96	(0.66, 1.38)	72,624	37	1.63	(0.99, 2.69)	47	1.26	(0.82, 1.92)
	III	49,252	37	0.94	(0.64, 1.39)	40	1.10	(0.75, 1.61)	83,929	27	1.11	(0.66, 1.89)	47	1.25	(0.82, 1.90)
	IV	48,509	44	1.08	(0.75, 1.57)	34	0.90	(0.60, 1.35)	117,442	56	1.64	(1.04, 2.59)	72	1.37	(0.93, 2.02)
	*p-value for trend*^b^			0.82			0.82				0.11			0.14	

HR = hazard ratio; CI = confidence interval

^a^Estimated using cox-proportional hazards regression adjusted for age (age (at randomization) and age2 or loess smoothed age-specific predicted quartiles) gender, body mass index, body mass index, education, cigarette smoking, alcohol use, and trial membership

^b^p-value of test for trend using either the number of teeth loss or the quartile of age-specific tooth loss as a continuous covariate

^c^Age-specific quartiles of tooth loss were estimated using loess regression

Shown in **Table 4** are the results of the analysis stratified on BMI. Among participants with a BMI ≥ 23.0 kg/m^2^, persons in the highest age-specific quartile of tooth loss had a significantly increased risk of liver cancer (HR = 1.71, 95%CI: 1.00, 2.92). Restricting the analysis to persons with a BMI ≥ 25.0 kg/m^2^ further strengthened the relationship (data not shown), however the estimates lacked precision as only 9.7% of the cohort had a BMI in this range. While the test for interaction with BMI was not significant (p_interaction_ = 0.28), the association between tooth loss and liver cancer was not evident among persons with a BMI < 23.0 kg/m^2^ (HR = 1.14, 95%CI: 0.82, 1.57). Similarly, there was no association between chronic liver disease mortality in either strata of BMI.

**Table 4:** Association of tooth loss to incident liver cancer and chronic liver disease (CLD) mortality by body mass index in the Nutrition Interventions Cohort, Linxian, China, 1986–2016.

		< 23.0 kg/m^2^							≥ 23.0 kg/m^2^						
			Liver cancer incidence			CLD mortality				Liver cancer incidence			CLD mortality		
Patient characteristic		Person-yrs	N	HR^a^	95% CI	N	HR^a^	95% CI	Person-yrs	N	HR^a^	95% CI	N	HR^a^	95% CI
Ever lost a tooth		** **													
	No	127,833	64	1.00	referent	62	1.00	referent	59,789	23	1.00	referent	30	1.00	referent
	Yes	341,592	209	0.99	(0.73, 1.35)	228	0.99	(0.73, 1.34)	122,755	74	1.43	(0.87, 2.37)	88	1.04	(0.66, 1.62)
Number of teeth lost		** **	** **	** **	** **	** **	** **	** **	** **	** **	** **	** **	** **	** **	** **
	0	127,833	64	1.00	referent	62	1.00	referent	59,789	23	1.00	referent	30	1.00	referent
	1–3	57,397	28	0.88	(0.56, 1.38)	27	0.86	(0.54, 1.35)	24,492	15	1.61	(0.84, 3.10)	6	0.45	(0.19, 1.09)
	4–6	76,203	45	1.06	(0.72, 1.56)	36	0.81	(0.54, 1.24)	30,922	13	1.06	(0.53, 2.12)	21	1.17	(0.66, 2.07)
	7–10	63,315	34	0.91	(0.59, 1.40)	51	1.26	(0.86, 1.86)	25,253	16	1.57	(0.81, 3.06)	19	1.15	(0.63, 2.09)
	11–20	71,245	47	1.05	(0.70, 1.58)	51	1.03	(0.69, 1.53)	22,404	14	1.45	(0.71, 2.98)	25	1.48	(0.83, 2.66)
	21–32	73,432	55	1.10	(0.73, 1.67)	63	1.11	(0.74, 1.66)	19,684	16	1.78	(0.86, 3.69)	17	1.04	(0.53, 2.04)
* *	*p-value for trend*^*b*^	* *	* *	*0*.*48*	* *	* *	*0*.*39*	* *	* *	* *	*0*.*22*	* *	* *	*0*.*65*	* *
Age-specific tooth loss^c^			** **												
	I	153,271	84	1.00	referent	81	1.00	referent	69,157	30	1.00	referent	39	1.00	referent
	II	93,436	65	1.11	(0.79, 1.54)	65	1.06	(0.76, 1.47)	36,972	27	1.47	(0.86, 2.51)	30	1.11	(0.68, 1.81)
	III	96,806	51	0.98	(0.69, 1.39)	67	1.21	(0.87, 1.68)	36,375	13	0.93	(0.48, 1.79)	20	0.94	(0.54, 1.63)
	IV	125,912	73	1.14	(0.82, 1.57)	77	1.10	(0.80, 1.52)	40,039	27	1.71	(1.00, 2.92)	29	1.20	(0.73, 1.97)
* *	*p-value for trend*^*b*^	* *		*0*.*58*	* *	* *	*0*.*42*	* *	* *	* *	*0*.*14*	* *	* *	*0*.*62*	* *

HR = hazard ratio; CI = confidence interval

^a^Estimated using cox-proportional hazards regression adjusted for age (age (at randomization) and age2 or loess smoothed age-specific predicted quartiles) sex, body mass index, education, cigarette smoking, alcohol use, and trial membership

^b^p-value of test for trend using either the number of teeth loss or the quartile of age-specific tooth loss as a continuous covariate

^c^Age-specific quartiles of tooth loss were estimated using loess regression

The results did not change when the analysis was restricted to persons ≥ 50 years of age or restricted to persons who never smoked (data not shown). The results also did not change when the first two years of follow-up were excluded or in a subset analysis of persons with known HBV/HCV status (data not shown). Similarly, exclusion of the persons in the Dysplasia Trial did not affect the results (data no shown).

## Discussion

In this prospective study in a rural Chinese population, persons in the highest quartile of age-specific tooth loss had an increased risk of liver cancer. The increased risk was more notable, and statistically significant, among women and among persons with a BMI > 23.0 kg/m^2^.

Three previous prospective studies have examined risk of liver cancer with tooth loss, one among a population of male Finnish smokers [15], one among patients at an oncology hospital in Japan [14], and one among community-dwelling octogenarians in Japan [13]. The study of Finnish smokers found significant associations between liver cancer and loss of 11–31 teeth (HR = 1.42, 95%CI: 1.01, 1.98), as well as loss of all teeth (HR = 1.45, 95%CI: 1.00, 2.10). The Japanese study in an oncology hospital found a significant association between liver cancer and loss of 12–23 teeth (HR = 1.74, 95%CI: 1.04, 2.89), but no association with loss of a greater number of teeth. The study of community-dwelling octogenarians found no significant association between liver cancer mortality and tooth loss (HR = 1.07, 95%CI: 0.98–1.17). No studies have prospectively evaluated the association of tooth loss with chronic liver disease mortality, however, several studies have found increased periodontal disease among persons with cirrhosis [28], and one study found decreased mortality among persons with cirrhosis treated for periodontal disease [29].

Why the associations between tooth loss and liver cancer varied among the studies is not certain, but several differences exist among the studies. The lack of association reported in the study of Japanese octogenarians may have been due to it being a small study of only 697 participants with a limited number of cancer deaths. In addition, liver cancer is more commonly diagnosed at ages younger than 80 years, so persons who live to be octogenarians are likely to be a healthier group of people who are at lower risk of cancer, overall. In the Japanese case-control study, the association seen with loss of 12–23 teeth, but not with the loss of a greater number of teeth, is curious, but as no further stratified analysis was presented, it is not clear whether the results differed by sex or other characteristics. In the study of male Finnish smokers, tooth loss of all levels was associated with a significantly increased risk of liver cancer. The lack of association among the men in the current study, in contrast to the Finnish study, could be due to a number of variables given that Finland, in contrast with Linxian, China is a high-income country with an economically-advantaged population. Nevertheless, in the current study, there was an association between the highest level of tooth loss and liver cancer among women. The association among women, but not among men, may be related to women having a higher prevalence of tooth loss (78.5% vs 72.1%) and being more likely to be in the highest quartile of age-specific tooth loss (31.2% vs 19.9%). Women were also more likely to have a BMI > 23.0 kg/m^2^, a factor that was also associated with higher risk of liver cancer associated with tooth loss.

Tooth loss can be the result of periodontal disease, but can also be the result of trauma or caries. A comprehensive oral health examination among ~600 participants of the cohorts, however, found that high levels of moderate to severe periodontal disease were present, while caries were less common [19]. These results suggest that the primary cause of tooth loss in the population was periodontal disease. Periodontal disease has been shown to be a risk factor for several systemic conditions, including cardiovascular disease [7], and stroke [8], and has been associated with cancers of the mouth [9], esophagus [10, 11], and gastrointestinal tract [11, 12]. These relationships may stem from the establishment of a systemic inflammatory condition through mediators such as histamine, cytokines and proteases [30], or alternatively, periodontal disease may serve as a marker for an immune system deficient in the ability to clear infection. Additionally, tooth loss may result from an oral flora that produces carcinogenic by-products.

Strengths of this study include its large sample size, prospective design, and extensive length of follow-up (up to 30 years). In addition, number of teeth lost, the primary exposure of interest, was determined by physical examination rather than self-report. Large variation in the primary exposure allowed robust estimation of a dose-response relationship. Methods for identification of cancer cases and causes of mortality assured essentially complete ascertainment of both outcomes. This population was highly homogeneous regarding occupation and socioeconomic status. We controlled for age using several methods, including categorizations based on loess smoothed age-specific predicted cut-offs. Further, we controlled for important risk factors for liver cancer including BMI, alcohol use, and cigarette smoking.

A limitation of this study was a lack of complete ascertainment of HBV/HCV status for the study population. The subset analysis of persons who were tested for HBV and HCV indicated, however, that the results were not affected by adjustment for viral status. The study also lacked information on exposure to aflatoxin B_1_, a known liver cancer risk factor. Previous research, however, reported low levels of aflatoxin B_1_ exposure in Linxian [31].

This study is the first to evaluate the effects of tooth loss on liver cancer incidence and chronic liver disease mortality in a rural, economically disadvantaged population. The increased risk of liver cancer with the highest levels of tooth loss, a relationship particularly evident among women, suggests that periodontal disease may be a risk factor for liver cancer. Further study of this relationship is clearly warranted as the identification of modifiable risk factors for liver cancer may have the potential to decrease the burden of the world’s second greatest contributor to cancer mortality.
